# A Zinc Finger Motif-Containing Protein Is Essential for Chloroplast RNA Editing

**DOI:** 10.1371/journal.pgen.1005028

**Published:** 2015-03-13

**Authors:** Tao Sun, Xiaowen Shi, Giulia Friso, Klaas Van Wijk, Stephane Bentolila, Maureen R. Hanson

**Affiliations:** 1 Department of Molecular Biology and Genetics, Cornell University, Ithaca, New York, United States of America; 2 Section of Plant Biology, Cornell University, Ithaca, New York, United States of America; University of North Carolina Chapel Hill, UNITED STATES

## Abstract

C-to-U editing of transcripts in plant organelles is carried out by small (<400 kD) protein complexes called editosomes. Recognition of the proper C target for editing is mediated by pentatricopeptide repeat (PPR) containing proteins that recognize cis-elements. Members of two additional gene families, the RIP/MORF and ORRM families, have each been found to be required for editing of particular sets of Cs in mitochondria and/or chloroplasts. By co-immunoprecipitation of the chloroplast editing factor ORRM1, followed by mass spectrometry, we have now identified a member of the RanBP2 type zinc fingers (pFAM00641) protein family that is required for editing of 14 sites in chloroplasts and affects editing efficiency of another 16 chloroplast C targets. In yeast two-hybrid assays, OZ1 (Organelle Zinc finger 1) interacts with PPR site recognition factors whose cognate sites are affected when *OZ1* is mutated. No interaction of OZ1 with the chloroplast editing factors RIP2 and RIP9 was detected; however, OZ1 interacts with ORRM1, which binds to RIP proteins, allowing us to build a model for the chloroplast RNA editosome. The RNA editosomes that act upon most chloroplast C targets are likely to contain a PPR protein recognition factor, either RIP2 or RIP9, ORRM1, and OZ1. The organelle zinc finger editing factor family (OZ) contains 4 members in Arabidopsis, three that are predicted to be targeted to chloroplasts and one to mitochondria. With the identification of OZ1, there are now 4 nuclear-encoded protein families known to be essential for plant organelle RNA editing.

## Author Summary

Transcripts encoding chloroplast and mitochondrial proteins of flowering plants are profoundly affected by RNA editing. In Arabidopsis, over 600 genomically-encoded Cs are modified to Us in organelle transcripts, altering the encoded amino acids and creating stop and start codons. Pentatricopeptide proteins are known to bind to cis-elements near C targets of editing and chloroplast RNA editing also requires members of two additional protein families. Nevertheless, not all protein components of the editosome have been identified. We now report the discovery of a member of fourth gene family essential for chloroplast RNA editing: OZ1, member of a family of Arabidopsis RanBP2-type zinc finger proteins. Identifying all of the proteins in the RNA editosome is critical for understanding the mechanism behind the remarkable specificity of C-to-U editing.

## Introduction

In vascular plants, specific cytidines are converted to uridines by RNA editing in the chloroplast transcripts [1–3]. A typical land plant modifies 30 to 40 C targets in chloroplasts, usually changing the encoded amino acid, and also acts upon hundreds of Cs in plant mitochondria [4,5]. The process is believed to be a correction mechanism to restore functional mRNAs in chloroplasts and mitochondria, whose genomes have undergone otherwise detrimental T-to-C changes [6].

The composition of the molecular machines that carry out plant organellar RNA editing, the editosomes, is not yet fully understood. Editosomes are found between the 200 and 400 kD markers on size exclusion columns [7]. Specificity of editing is achieved through the recognition of a *cis*-element 5’ adjacent to the editable cytidine by a pentatricopeptide repeat (PPR) motif-containing protein [8–10]. Recognition codes that match particular PPRs with nucleotides within the bound RNA region have been proposed [11,12]. Multiple PPRs in editing factors are followed by a so-called E domain and many PPR protein editing factors also contain a C-terminal DYW domain [13]. The DYW domain exhibits sequence motifs characteristic of cytidine deaminases [14] and is required for editing activity of some PPR-DYW proteins but is dispensable in others [15–20]. Attempts to demonstrate deaminase activity of purified DYW domains have failed so far [19,21]. Nevertheless, it remains possible that a DYW domain providing an enzymatic activity needed to deaminate cytidine to uridine could be present in all editosomes even if not on all PPR recognition factors. A protein named DYW1, which lacks any PPRs, was found to be required for editing of a chloroplast C target that is recognized by a PPR-E factor that lacks a DYW domain [22]. Mutating conserved residues characteristic of deaminases in DYW1 or in the DYW domains of QED1 and RARE1 results in impaired editing [23,24].

In addition to the large PPR protein family that provides site-specific recognition, members of two other plant protein families have been identified as components of editosomes, the RIP/MORF family and the ORRM family [7,25,26]. As each of these additional proteins are needed for efficient editing of some C targets but not others, editosomes that act upon particular C targets differ not only in the site-specific PPR protein recognition factor they contain, but also in which members of these additional families comprise the protein complex. The chloroplast editing factor ORRM1 contains both a RIP domain and an RRM (RNA Recognition Motif) domain [25]. The ORRM1 protein belongs to a distinct clade of RRM-containing proteins, and the RRM domain by itself is able to provide RNA editing activity to *orrm1* mutants [25].

In order to identify components of chloroplast editosomes that contain ORRM1, *Arabidopsis thaliana orrm1* mutants were complemented with an epitope-tagged ORRM1 protein. A candidate ORRM1-interacting protein, encoded by At5g17790, was identified in immunoprecipitates. Through analysis of mutant and silenced tissue, we demonstrated that the candidate protein is a novel chloroplast editing factor. The protein, which we have named OZ1 (Organelle Zinc finger 1), belongs to the RanBP2 type zinc finger protein family, and is required for editing of 14 sites in chloroplasts and affects editing efficiency of another 16 chloroplast C targets. OZ1 is a member of an Arabidopsis protein family that encodes three additional proteins predicted to be targeted to chloroplasts or mitochondria. Identification of OZ1 as a chloroplast editing factor implicates a previously unsuspected class of zinc finger-containing proteins as potentially involved in RNA editing or other aspects of plant organelle RNA metabolism.

## Results

### N-terminal tagging of ORRM1 preserves editing activity

Preliminary experiments demonstrated that an epitope tag placed at the C-terminus of ORRM1 disrupted its function (S1 Fig A and B). We therefore produced an ORRM1 expression vector with a RecA transit sequence followed by three tandem FLAG tags fused with a strepII tag (3FS tag) (S1 Fig A and C). This construct, designated, RecA-3FS-mORRM1, resulted in significant increase of editing of *matK* C640, from 11% to 20%, following transfections of *orrm1* protoplasts (S1C Fig). The low complementation level can be largely attributed to the size of the vector used in this assay. While the plasmid harboring RecA-RRM is around 6kb (S1 Fig), the N-terminal tagged ORRM1 is integrated into a binary vector around 14kb, and plasmids over 10 kb are known to exhibit lower transfection efficiency [27].

**Fig 1:**
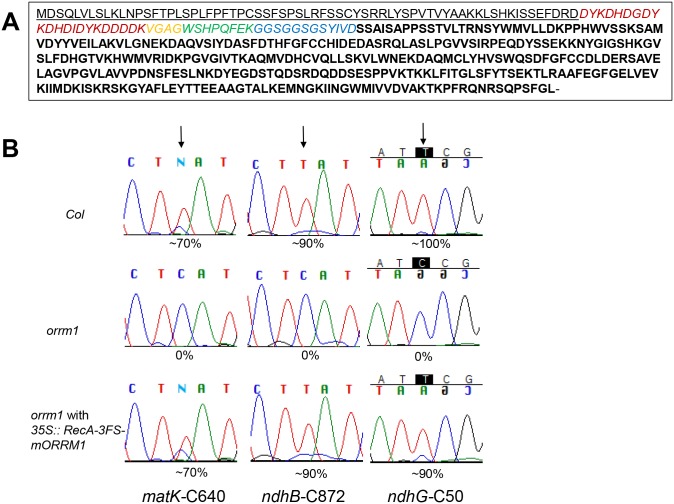
Stable integration of 35S::RecA-3FS-mORRM1 into the *orrm1* mutant restores normal editing level in plastids. (A) Protein sequence of RecA-3FS-mORRM1. Transit peptide sequence from RecA is underlined. Sequence of epitope tags is italic. 3xFLAG, spacer, StrepII and Glycine-Serine linker are labeled with red, yellow, green and blue, respectively. Sequence of mature ORRM1 without the 54 amino acid transit peptide is bolded. (B) Portion of electrophoretograms from RT-PCR bulk sequencing of *matK* C640, *ndhB* C872 and *ndhG* C50 is shown for the *Columbia* wild-type, *orrm1*, and a stable transformant expressing RecA-3xFLAG-strepII-mORRM1 under control of a 35S promoter. The editing sites are indicated with arrows. The complementary strand of the sequenced *ndhG* is shown, as sequencing was done from a reverse direction.

We investigated whether the epitope-tagged protein (Fig. 1A) could restore editing in transgenic plants obtained by root transformation of *orrm1* mutant plants. Transgenic plants of normal phenotype were obtained and RNA was extracted for use in editing assays. Editing extent of *matK* C640, *ndhB* C872 and *ndhG* C50, which exhibit decreased editing in *orrm1*, was examined by bulk sequencing (Fig. 1B). Editing of all three sites was restored to wild-type level in the RecA-3FS-mORRM1 transgenic plants.

### Co-immunoprecipitation of ORRM1 followed by mass spectrometry identifies a candidate interacting protein

Total leaf proteins were used to perform ORRM1 immunoprecipitation (IP). Wild-type Arabidopsis, *Columbia* ecotype, was included as a negative control for comparison in order to eliminate non-specific binding proteins. We observed that the affinity of the strepII tag on ORRM1 to streptactin resin was poor, which was probably due to its internal position caused by the N-terminal fusion of the FLAG tag (Fig. 1A). Therefore we used only anti-FLAG antibodies for immunoprecipitation.

**Fig 2:**
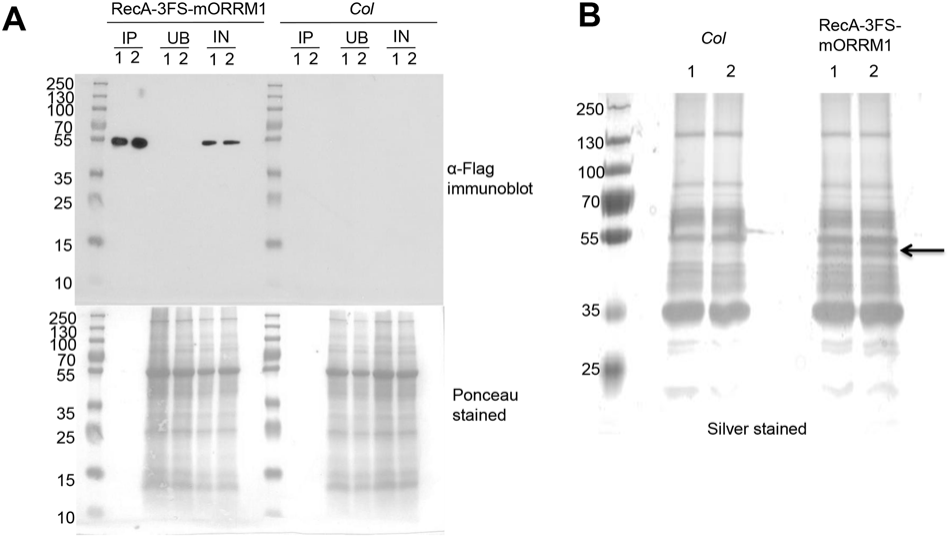
Immunoprecipitation of 3FS-ORRM1 using anti-FLAG resins. Two independent IP experiments were performed for each group. (A) Immunoblot of immunoprecipitate (IP), unbound flowthrough (UB) and total input (IN) for both *Col* and transgenic RecA-3FS-mORRM1. 10 μg total protein loaded for IN and UB. 1% of the IP was loaded. Anti-FLAG-HRP was used to detect FLAG-tagged protein. (B) 10% of the IP was separated by 10% Tris-Glycine SDS-PAGE and silver stained. Arrow indicates location of tagged ORRM1.

As is shown in Fig. 2, the anti-FLAG antibody recognizes one band from the transgenic plant samples, but none in the wild-type sample. The unique band’s electrophoretic mobility is slightly slower than that expected for the predicted 42 kD size of the tagged ORRM1, possibly due to post-translational modifications. Anti-FLAG resins retained almost all tagged ORRM1 protein from the extract (Fig. 2A). The elutions from both ORRM1 and negative control were separated by a SDS-PAGE gel and silver stained. The bait, 3FS-mORRM1, is clearly seen in the transgenic plant IP but missing in the *Col* negative control (Fig. 2B). The immunoprecipitates were subjected to MS/MS mass spectrometry in order to identify ORRM1-binding proteins. The protein encoded by At5g17790 was selected for further investigation because after the ORRM1 peptides, it had the largest number of matches in MS/MS spectra and was not detected in the negative controls. S1 Table describes the peptides detected that resulted in the identification of the At5g17790 as a candidate ORRM1-interacting protein.

### Characterization of mutants in At5g17790

**Fig 3:**
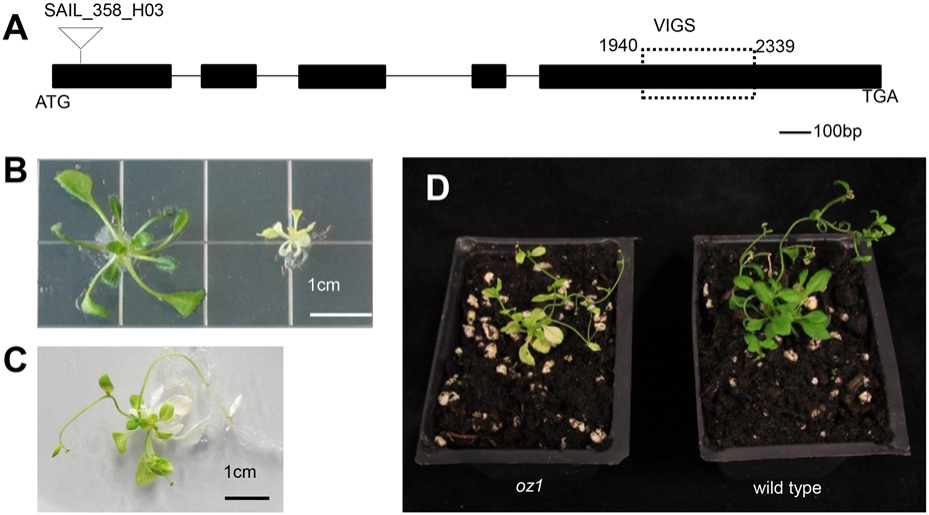
*OZ1* (At5g17790) gene structure and mutant phenotype. (A) Gene structure of *OZ1*. Triangle indicates the location of the T-DNA. Dashed box indicates the gene specific region selected for VIGS (Virus Induced Gene Silencing). (B-D) *oz1–1* phenotype. (B) plants grown on MS media for 4 weeks. Left, wild type sibling, right, homozygous *oz1* mutant. (C) new leaves turned light green on a six week old *oz1* mutant. (D), eight week old *oz1–1* (left) grows in soil compared to wild type (right).

At5g17790 contains two tandem C2X10C2 zinc finger domains [28] called RanBP2 type zinc fingers (X2GDWICX2CX3NFARRX2CXRCX2-PRPEX2; pFAM00641), which were characterized in the Ran Binding Protein 2 (RanBP2). Ran is a small GTPase and RanBP2 is a nucleoporin that binds Ran via the zinc finger motifs. This gene previously was identified as mutated in a variegated Ds insertional mutant of *Arabidopsis thaliana* Landsberg erecta, but the cause of the chloroplast developmental aberration was not determined [28]. We obtained one T-DNA insertional line in *A*. *thaliana* ecotype Columbia from ABRC, SAIL_358_H03 (Fig. 3A). In contrast to the mutant in the Landsberg ecotype, the homozygous Columbia mutant showed a uniform yellow phenotype as a young seedling, as shown in Fig. 3B. Subsequent growth on sucrose media result in the appearance of light green, non-variegated leaves (Fig. 3C). These older mutant seedlings could be transferred to soil, where the pale green leaves were able to support autotrophic growth (Fig. 3D). The protein encoded by At5g17790 was given the name OZ1 (Organelle Zinc finger 1).

### *OZ1* mutation leads to altered editing at most chloroplast sites

**Fig 4:**
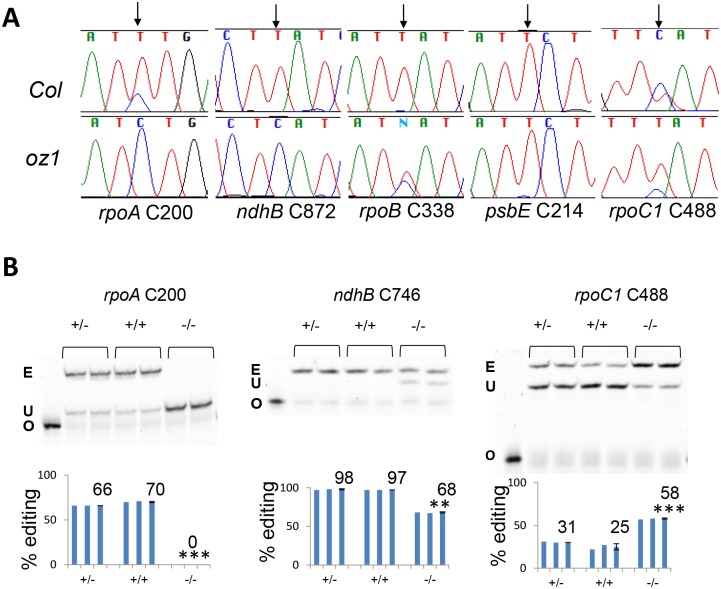
RNA editing at multiple plastid sites is affected in *oz1*. (A) Editing of plastid sites is disrupted or enhanced in *oz1* as demonstrated by bulk sequencing. Portion of electrophoretograms from RT-PCR bulk sequencing is shown. Wild-type *Columbia*, upper lane. *oz1*, lower lane. Arrows indicate the position of the editable C. (B) Editing extent is examined by Poisoned Primer Extension assay. Oligonucleotides (O) were loaded in the first lane for each gel figure. +/-, heterozygote; +/+, wild type *Col*;-/-, homozygous *oz1*. E, edited band; U, unedited band. Significance * (P<0.05), ** (P<0.01), *** (P<0.001)

RNA from 4-week-old *oz1–1* homozygous mutants and the siblings was extracted and the editing extent was examined by bulk sequencing as shown in Fig. 4A. The *oz1* mutation causes altered editing of various chloroplast C targets. For example, editing of *rpoA* C200 and *ndhB* C872 is completely lost in *oz1–1* while editing of *rpoB* C338 is partially disrupted (Fig. 4A). No obvious effect was observed on *psbE* C214 editing in the *oz1–1* mutant. On the contrary, at *rpoC1* C488, the mutant editing level is up-regulated compared to the wild-type (Fig. 4A).

Because poisoned primer extension (PPE) is a more sensitive method to measure editing extent than bulk sequencing [4,7], all chloroplast editing sites were assayed both in *oz1* and its siblings using PPE (Fig. 4B and Table 1). The *oz1–1* allele is clearly recessive because no significant editing difference is seen between heterozygotes and wild-type plants. Editing of *rpoA* C200 is 0% in *oz1–1* by PPE, which confirmed the result from bulk sequencing. *ndhB* C746 editing dropped from 97% in wild-type to 68% in the mutant. Editing of *rpoC1* C488 increases from 25% in wild type to 58% in the mutant, in agreement with the bulk sequencing data.

**Table 1:** Chloroplast editing extent in *oz1–1* and *orrm1* measured by PPE.

Editing site	Editing PPR protein	Editing extent in *oz1–1*%	Editing extent wild type level %	Editing level change	Editing level change
				*oz1–1*:: WT	*orrm1*:: WT
*rpoC1* C488	DOT4	72±2	24±2	**+200%**	0
*rps12-(i1)* C58	OTP81/QED1	0±0	28±2	**-100%**	**-99%**
*ndhD* C2	CRR4	0±0	56±4	**-100%**	**-57%**
*ndhB* C836	OTP82	0±0	95±0	**-100%**	**-95%**
*ndhG* C50	OTP82	0±0	84±3	**-100%**	**-94%**
*rpoA* C200	CLB19	0±0	71±3	**-100%**	**-73%**
*clpP* C559	CLB19	0±0	61±1	**-100%**	**-73%**
*ndhB* C1255	CREF7	0±0	99±0	**-100%**	**-60%**
*ndhB* C872	QED1	0±0	90±6	**-100%**	**-99%**
*ndhB* C467	CRR28	0±0	84±3	**-100%**	**-95%**
*accD* C1568	QED1	2±3	77±0	**-97%**	**-99%**
*ndhB* C586		5±1	92±1	**-95%**	**-95%**
*ndhD* C878	CRR28	5±0	85±2	**-94%**	**-97%**
*ndhB* C830	ELI1	7±1	97±2	**-93%**	**-71%**
*ndhB* C726		2±0	22±3	**-91%**	**-73%**
*matK* C640	QED1	12±2	85±1	**-86%**	**-97%**
*ndhD* C674	OTP85	16±1	91±4	**-82%**	**-99%**
*accD* C794	RARE1	27±6	95±4	**-72%**	0
*ndhD* C887	CRR22	33±0	83±4	**-60%**	**-96%**
*rpoB* C2432	QED1	44±2	82±1	**-46%**	**-92%**
*petL* C5		45±1	78±1	**-42%**	-1%
*rpoB* C551	CRR22	60±0	92±0	**-35%**	**-75%**
*ndhB* C746	CRR22	68±1	98±0	**-31%**	**-52%**
*rpl23* C89	OTP80	67±4	83±1	**-22%**	0
*ndhF* C290	OTP84	80±0	97±0	**-18%**	0
*rpoB* C338	YS1	76±3	91±0	**-16%**	**-14%**
*ndhB* C149		80±0	95±0	**-11%**	0
*psbF* C77	LPA66	88±1	97±1	**-9%**	0
*ndhB* C1481	OTP84	85±1	90±2	**-6%**	0
*psbZ* C50	OTP84	87±0	92±1	**-5%**	0
*atpF* C92	AEF1	90±0	93±2	-3%	0
*rps14* C149	OTP86	93±2	90±1	-3%	**-37%**
*ndhD* C383	CRR21	96±1	98±1	-2%	0
*rps14* C80		93±1	92±1	-1%	0
*psbE* C214	CREF3	98±0	98±2	0	0
*ndhB* C708		0	0	0	**-99%**
*ndhB* C153		n/a	n/a	0	**-10%**

Known PPR factors are listed next to C targets for which they are required. + indicates increased editing extent compared to wild type;—indicates decreased editing extent. n/a, not assayed. Editing extent that is significantly (P<0.05) different from wild type level was bolded. Table from reference [26] was modified with additional editing PPR proteins [16][24][40][41].

The assay data for the complete set of chloroplast sites is shown in Table 1. 14 sites have major loss of editing (>90% decrease in editing) in *oz1–1* and 15 other sites have significantly decreased editing (>5%, P<0.05). Although editing defects are massive, editing events on the same transcript are not all affected in the same pattern by *oz1*, hence the editing defects are unlikely to be a secondary effect caused by some change in a transcript itself. For example, *ndhD* C2 and *ndhD* C878 lost over 90% of the wild-type editing extent in *oz1–1*, but *ndhD* C383 is not affected at all. On the contrary, the sites recognized by the same PPR protein are largely affected in the same way by *oz1–1* mutation. *ndhD* C878 and *ndhB* C467 share the same PPR recognition factor CRR28 and both of them lose over 90% editing in *oz1–1* (Table 1). Likewise, editing of both *ndhB* C836 and *ndhG* C50 (controlled by OTP82) and editing of both *rpoA* C200 and *clpP* C559 (recognized by CLB19) are similarly affected. *ndhF* C290 *ndhB* C1481 and *psbZ* C50 are all recognized by OTP84, and in *oz1–1*, all sites exhibit mild defects in editing (5%-20%) (Table 1).

Given that OZ1 is immunoprecipitated by ORRM1, we also compared the *OZ1*-dependent sites and *ORRM1*-dependent sites to examine if these two factors participate in the same editing events. Indeed, editing efficiencies of the 14 *OZ1-*dependent sites are all severely affected in the *orrm1* mutant. Many other sites mildly affected by the *oz1–1* mutation are also *orrm1*-dependent (Table 1). 8 sites are controlled only by OZ1 but not by ORRM1. Taken together, OZ1 is a genuine editing factor for the majority of C targets in chloroplasts.

### Transient silencing of *OZ1* leads to chloroplast editing defects

**Fig 5:**
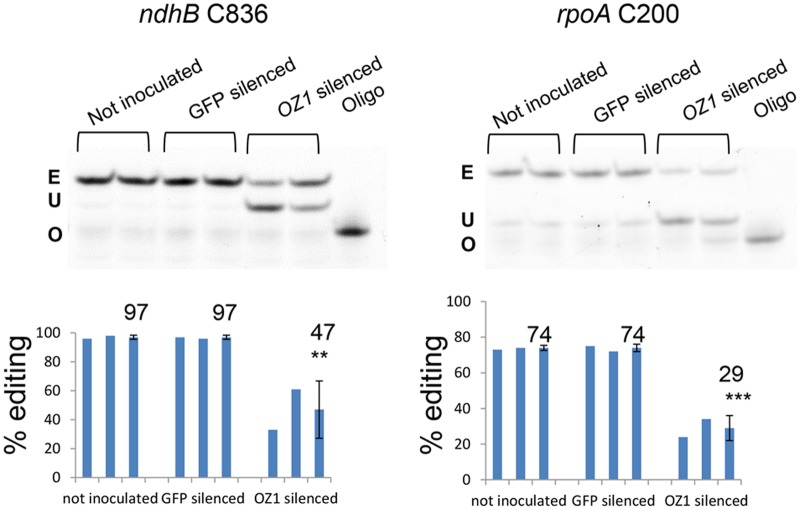
Transient silencing of *OZ1* in Arabidopsis results in chloroplast editing defects. Two replicates for each treatment were assayed by PPE. Not inoculated, untreated plants. GFP silenced, inoculated with Agrobacteria harboring a GFP silencing construct. *OZ1*-silenced, plants that were inoculated with Agrobacteria harboring a *GFP* and *OZ1* co-silencing construct. Average for each group is displayed in a third bar. E: edited band, U: unedited band, O: oligonucleotide. Significance ** (P<0.01), *** (P<0.001)

Since a second T-DNA mutant in the coding region of *OZ1* was not available, we performed Virus Induced Gene Silencing (VIGS) to transiently silence *OZ1* expression in young Arabidopsis seedlings. To monitor the silencing efficiency, a GFP co-silencing marker harbored in the VIGS construct was used [29]. Agrobacteria carrying either the OZ1/GFP co-silencing construct or the GFP silencing construct alone were inoculated into 2 week-old 35S::GFP expressing Arabidopsis seedlings. After growth in long days for 5 more weeks, the editing extents in RNA from GFP-silenced leaves and from uninoculated plants were analyzed by PPE. There were no differences between leaves of GFP-silenced plants and untreated plants (Fig. 5). *ndhB* C836 editing extent decreased from 97% in the untreated control to 47% in *OZ1* silenced plants (P<0.01). *rpoA* C200 editing extent dropped from 74% in untreated control to 29% in *OZ1* silenced plants (P<0.001). These results agree with the data from *oz1–1* mutants, in which editing is abolished at both sites. The residual editing in the silenced plants is probably caused by incomplete depletion of OZ1 protein.

### Transient expression of *OZ1* in *oz1–1* protoplasts complements the editing defects

Although the young *oz1–1* mutant has a severely defective phenotype, the plants gradually recover some chlorophyll. In a 6-week- old *oz1–1* plant, the old leaves remain pale yellow while the new leaves are light green (Fig. 3C). To investigate whether editing defects are rescued in the light green leaves, editing of RNA extracted from both the pale yellow leaves and the light green leaves were analyzed by bulk sequencing.

**Fig 6:**
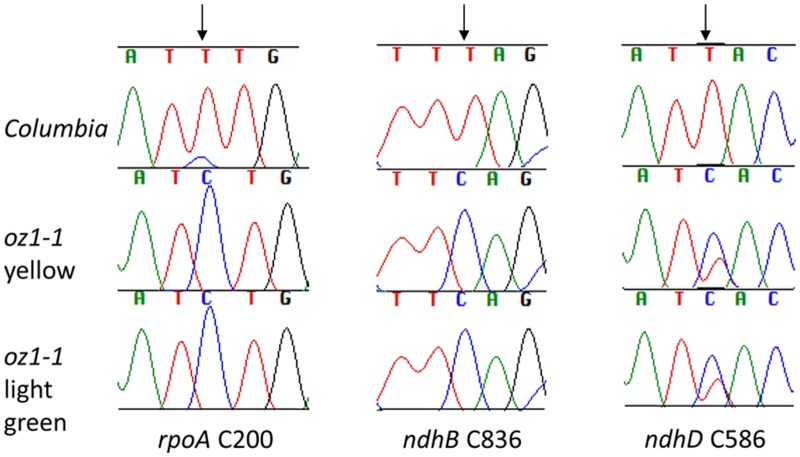
Chloroplast editing is not recovered in light green leaves of *oz1–1*. RNA from yellow leaves and light green leaves of 8-week-old *oz1–1* plants were used for RT-PCR and bulk sequencing. Portions of electrophoretograms are shown. Arrow indicates the position of editable C target.

Although pigmentation has been partially recovered in light green leaves, plastid editing is still defective in those leaves compared to wild type *Col* (Fig. 6). No obvious difference in editing between yellow and green leaves was observed. This finding indicates that the defects in editing are not due to a pleiotropic effect caused by some other chloroplast developmental problem in yellow chlorophyll-deficient leaves. Green leaves were therefore used to prepare protoplasts. *OZ1* was cloned into a pSAT4a vector to create 35S::*OZ1*, a plant transient expression vector driven by a 35S promoter for transfections of *oz1–1* protoplasts. A chloroplast targeted YFP construct (35S::*cpYFP*) was included as a negative control. Monitoring of transfection efficiency of the YFP constructs by microscopy indicated expression of YFP in over 50% of the protoplasts.

**Fig 7:**
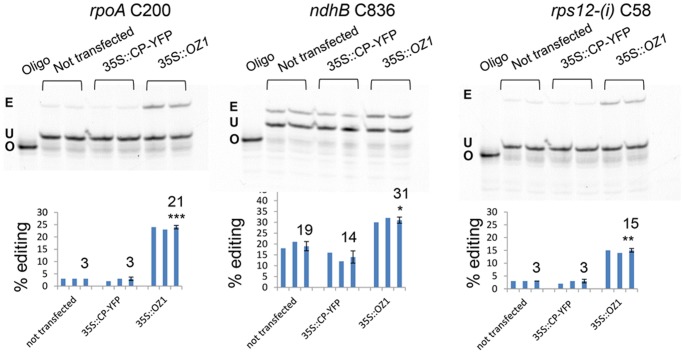
Transient expression of *OZ1* under a 35S promoter in *oz1–1* mutant protoplasts complements the editing defects. Two repeats of each treatment were assayed by PPE. The average for each group is displayed in a third bar. Not transfected: untreated *oz1–1* mutant protoplasts. 35S::CP-YFP: *oz1–1* protoplasts transfected with 35S::*cpYFP*. 35S::OZ1: *oz1–1* protoplasts transfected with 35S::*OZ1*. E, edited band; U, unedited band; O, oligonucleotide. Significance * (P<0.05), ** (P<0.01), *** (P<0.001).

RNA was extracted from protoplasts two days after the transfection and analyzed by PPE to examine the editing efficiency (Fig. 7). No significant difference in editing was seen between the untransfected control and the 35S::*cpYFP*-transfected control. Introduction of 35S::*OZ1* significantly increases the editing level for all the sites we tested. *rpoA* C200 increased from 3% to 21%, *ndhB* C836 from 19% to 31% and *rps12-(i1)* C58 from 3% to 15%. This confirms that the editing defects in *the oz-1–1* mutant can be reduced by introduction of *OZ1*.

### Stable expression of *OZ1* in *oz1–1* mutant plants also complements the editing defects

**Fig 8:**
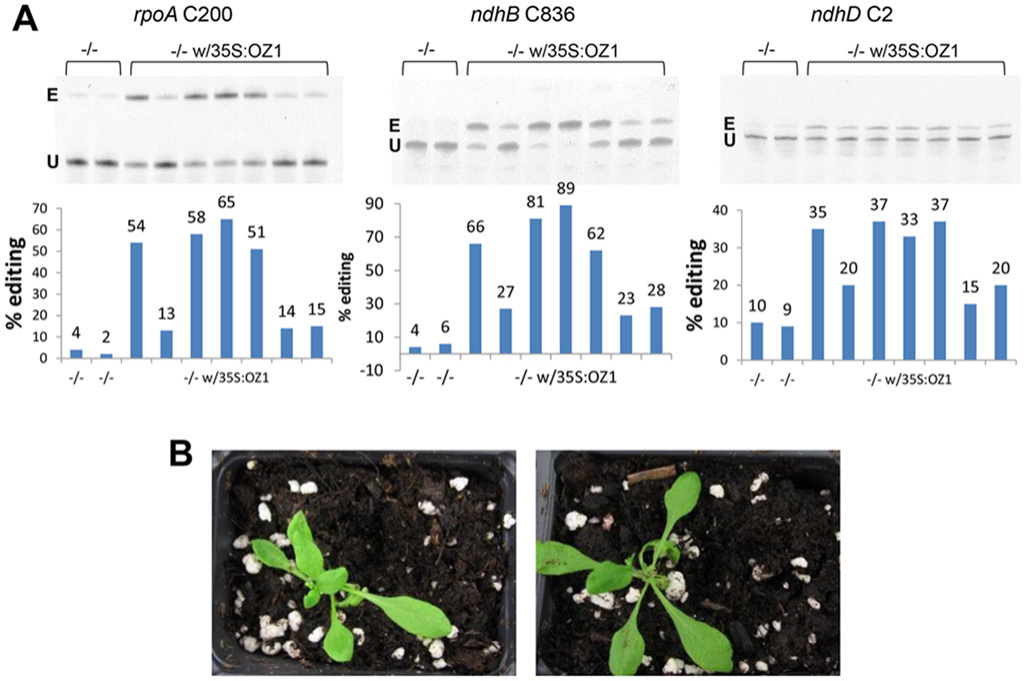
Stable expression of *OZ1* under a 35S promoter in *oz1* mutant plants complements the editing and phenotypic defects. (A) Three different C targets of editing in several different transgenic plants were assayed by Poisoned Primer Extension (PPE). PPE bands were quantified by ImageQuant software and illustrated in graphs.-/-, homozygous *oz1* mutant plants;-/- w/35S:OZ1, transgenic *oz1* mutant plants transformed with a construct expressing OZ1 driven by 35S promoter. E, edited band; U, unedited band. (B) independent 9-week-old transgenic mutant plants lacking the yellow phenotype observed in the mutant plant.

Because of the poor growth of the homozygous *oz1* mutant plant (Fig. 3D), we decided to transform the heterozygous plant by floral dipping with a construct expressing *OZ1* under the control of a 35S promoter. Genotyping the transgenic plants growing on a selectable plate allowed us to recover several independent plants homozygous mutant for the endogenous *oz1* alleles but expressing the *OZ1* transgene. The introduction of a functional OZ1 complements the editing defect in all the transgenic plants assayed (Fig. 8A). Positional effects on the transgene are known to affect expression and likely resulted in the range of responses in the transformed plants. For example, in different plants, *rpoA* C200 editing extent ranged from 13%-65% (Fig. 8A). The restoration of editing extent in some transgenic mutant plants is much more pronounced than with the transient expression in the *oz1* mutant protoplasts, e.g. 89% vs. 31% for *ndhB* C836, and reaches almost the level observed in the wild-type plant. In addition to reverting the editing defects, the introduction of OZ1 *in planta* also suppress the yellow phenotype observed in the mutant plant (Fig. 8B). The reversion of both editing and phenotypic defects by expression of a functional OZ1 demonstrates the role of this protein in both phenomena.

### OZ1 interacts with ORRM1

**Fig 9:**
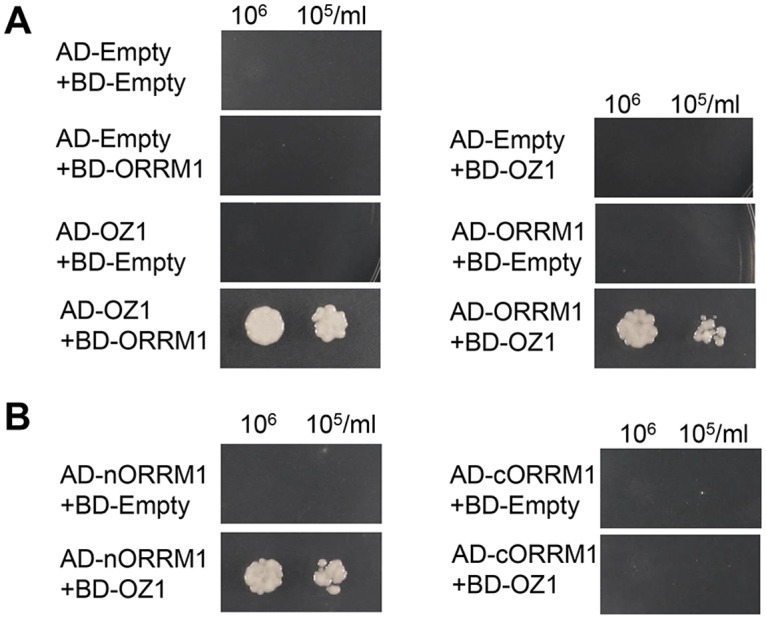
Yeast two-hybrid assay of interaction of ORRM1 and OZ1. (A) OZ1 interacts with ORRM1 in the yeast two-hybrid assays. (B) N-terminus of ORRM1 mediates the interaction with OZ1. AD-Empty, pGADT7 empty vector. BD-Empty, pGBKT7 empty vector. Yeast single transformants were mated to make double transformants in order to test interactions. Yeast were grown in-leucine-tryptophan double-dropout media overnight before they were harvested and diluted into cell density 10^6^/ml and 10^5^/ml. 10μl of each dilution was spotted onto the-leucine-tryptophan—histidine—adenine quadruple dropout plates. Pictures were taken 3 days after inoculation. nORRM1: amino acids 55–274 ORRM1, which contains the RIP-RIP domain. cORRM1: amino acids 275–374 of ORRM1, which contains the RRM domain.

A yeast two-hybrid (Y2H) assay was employed to examine the interaction between OZ1 and ORRM1. Both OZ1 and ORRM1 are plastid-targeted proteins, so the predicted transit peptide sequences were removed from each before cloning them into AD/BD fusion constructs. As shown in Fig. 9A, OZ1 interacts with ORRM1 in yeast. The interaction is not affected by the position of the fusion protein since both AD-OZ1/BD-ORRM1 and its reciprocal pair AD-ORRM1/BD-OZ1 showed interaction, implicating a genuine interaction between these two proteins. ORRM1 was further divided into nORRM1 and cORRM1, encompassing the RIP-RIP and the RRM domain respectively. nORRM1 but not cORRM1 interacts with OZ1, indicating the RIP-RIP domain actually mediates the interaction with OZ1 (Fig. 9B).

### OZ1 interacts with other components of chloroplast editosomes

**Fig 10:**
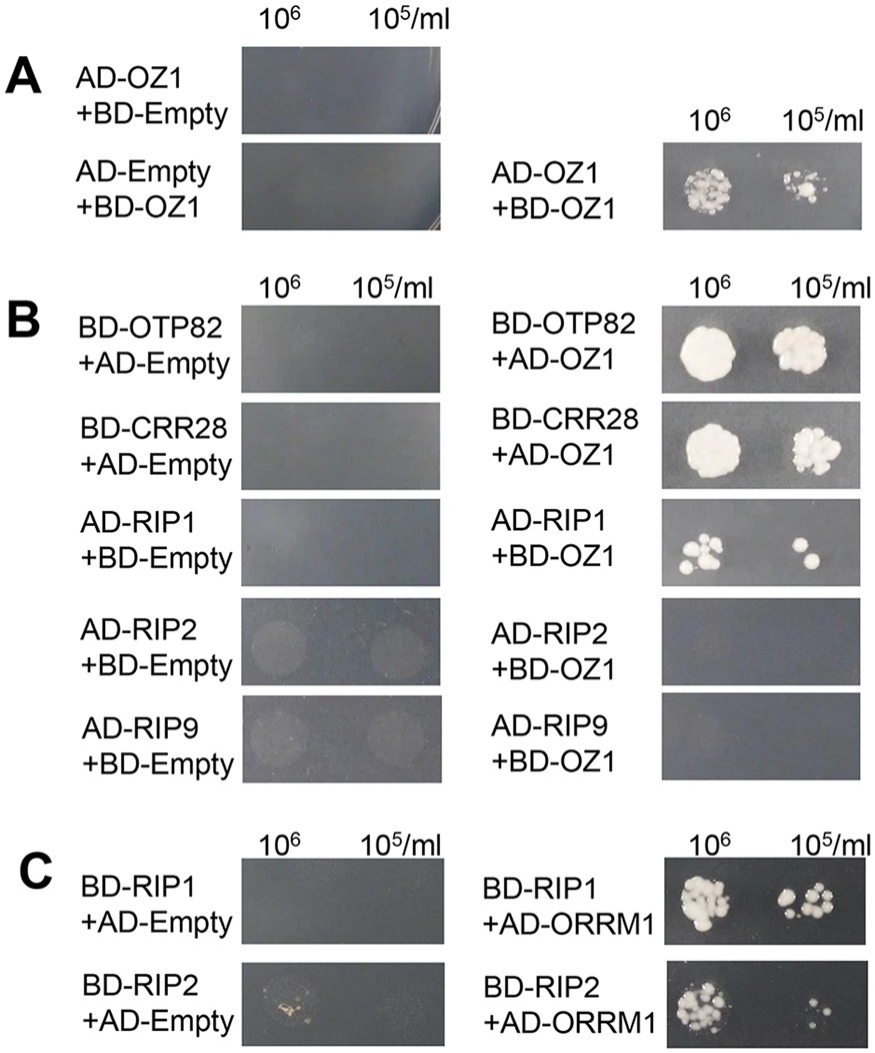
OZ1 interacts with other components of the editing complex. (A) OZ1 dimerizes (B) OZ1 interacts with OTP82, CRR28 and RIP1 but not RIP2 or RIP9. (C) ORRM1 interacts with RIP1 and RIP2. All interactions were tested on—Leu-Trp-His-Ade dropout media. Image taken 3 days after spotting of strains.

We suspected that OZ1 might also interact with other components of chloroplast editosomes in addition to ORRM1, such as additional PPR site recognition factors and members of the RIP/MORF protein family. In order to determine whether OZ1 can dimerize and/or interact with other components of the editing complex, we performed a series of Y2H assays. OZ1 fused to either AD or BD does not show any auto-activation for HIS and ADE reporters, while yeast with AD-OZ1/BD-OZ1 is able to grow on histidine and adenine deficient media, indicating self-interaction (Fig. 10A). OZ1 also interacts with OTP82 and CRR28, as shown in Fig. 10B, a result expected from the effect of the *oz1–1* mutation on C targets controlled by OTP82 and CRR28 (Table 1). OZ1 exhibits a weaker interaction with RIP1; fewer colonies are seen in the RIP1/OZ1 combination (Fig. 10B). However, no interaction was observed between OZ1 and RIP2 or RIP9 (Fig. 10B), even though RIP2 and RIP9 are essential for editing of a large number of chloroplast C targets. We considered the possibility that OZ1 associates with RIP2 and RIP9 via ORRM1. To test this hypothesis, we performed a Y2H assay for ORRM1 and RIP proteins (Fig. 10C). Both RIP1 and RIP2 can interact with ORRM1. RIP9 fused with the GAL4 binding domain strongly autoactivates *HIS* and *ADE* reporters, so RIP9 was not tested in this experiment. ORRM1 with the GAL4 activation domain shows no autoactivation (Fig. 9A). Our data is consistent with ORRM1 as a mediator of interaction between OZ1 and RIP2 within the chloroplast editosome.

### OZ1 belongs to a small family in Arabidopsis

**Fig 11:**
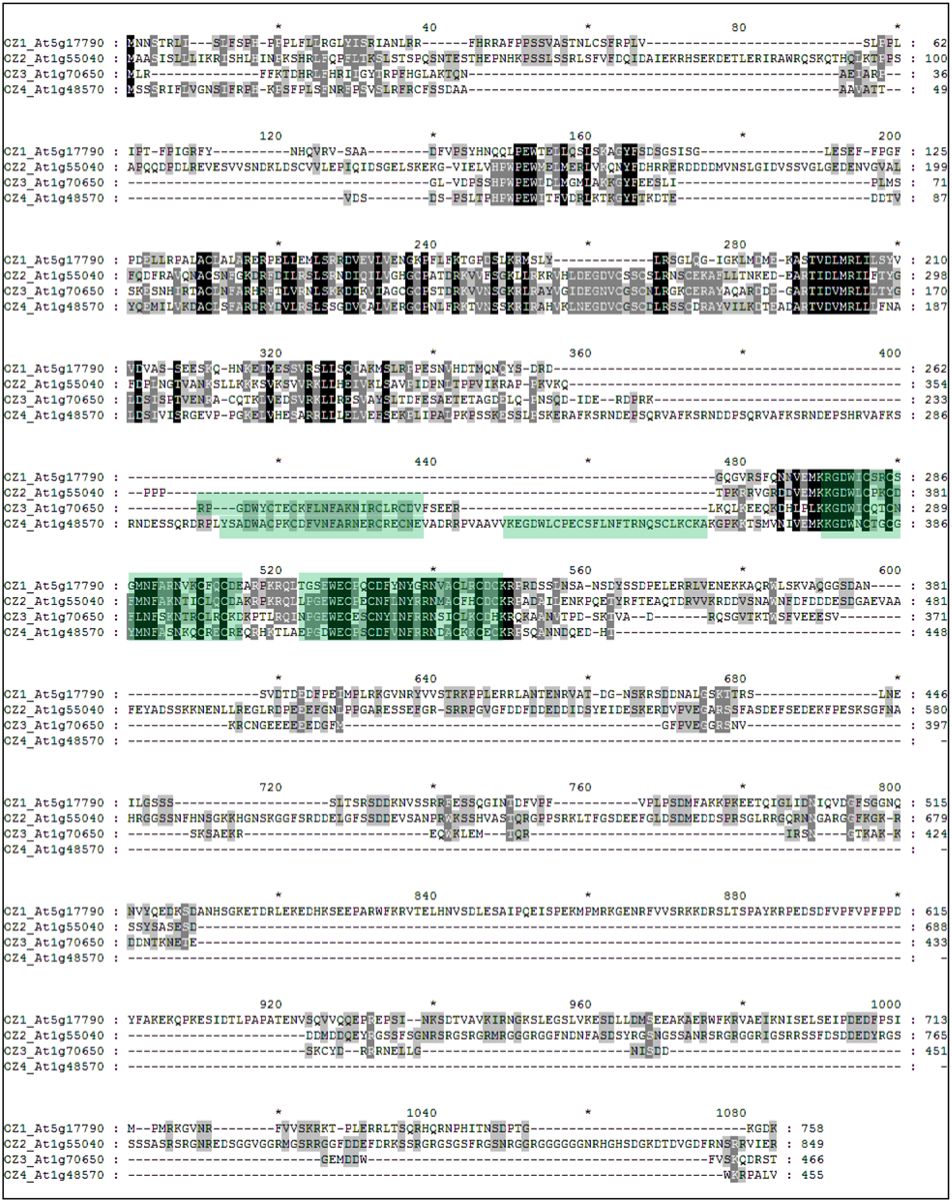
OZ1 belongs to a small family in Arabidopsis. Protein sequence of OZ1 family members are aligned by T-coffee [39] and then visualized by GeneDoc. Conserved regions are shaded in black, dark grey, and grey according to the conserved percent (100, 80, 60). Predicted zinc finger motifs are colored in green. OZ family members have various numbers of zinc finger motifs.

Three highly similar RanBP2 zinc finger proteins were found in Arabidopsis protein database in a BLAST search with OZ1. In 2004, these proteins were reported to comprise a four-member gene family of unknown function [28]; however, changes in gene models result in a new alignment (Fig. 11). The protein sequence alignment by T-coffee shows presence of multiple highly conserved regions in the N-terminal portion of the protein, past the predicted transit sequences, with various numbers of zinc finger motifs and more variable C-terminal regions (Fig. 11).

**Fig 12:**
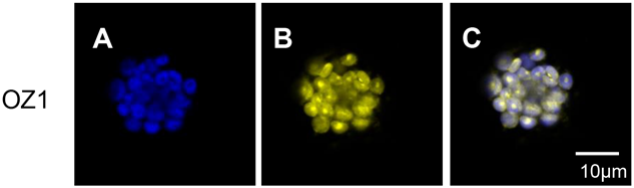
Localization of YFP fusion to OZ1 N-terminal sequence at punctuate loci in chloroplasts. Leaf protoplasts from Arabidopsis L*er* were transfected with a construct encoding the first 100 amino acids of OZ1 fused with YFP under the control of a tandem CaMV 35S promoter. Protoplasts were examined for fluorescence 3 days after transfection. (A) Chlorophyll autofluorescence is marked as blue. (B) YFP is yellow. (C) Merge of chlorophyll and YFP signals. (A-C) OZ1 is targeted to chloroplasts.

In order to investigate the subcellular location of OZ1, we fused the N-terminal sequences encoding 100 amino acids to yellow fluorescent protein (YFP) and transfected protoplasts. Confocal microscopic imaging revealed that OZ1-YFP is located in the chloroplasts at punctate loci (Fig. 12). Previously, the entire coding region of OZ1 (VAR3) was fused to GFP and observed to be located within chloroplasts at punctate loci [28]. OZ1 was also detected in chloroplast nucleoid preparations by mass spectrometry (ppdb.tc.cornell.edu). In addition, Target P predicts all of the other OZ1 family members to be organelle targeted [30], one in mitochondria and two in plastids (S2 Table). Proteomics studies have also found OZ4 (At1g48570) in both chloroplast nucleoids and stroma (ppdb.ts.cornell.edu).

**Fig 13:**
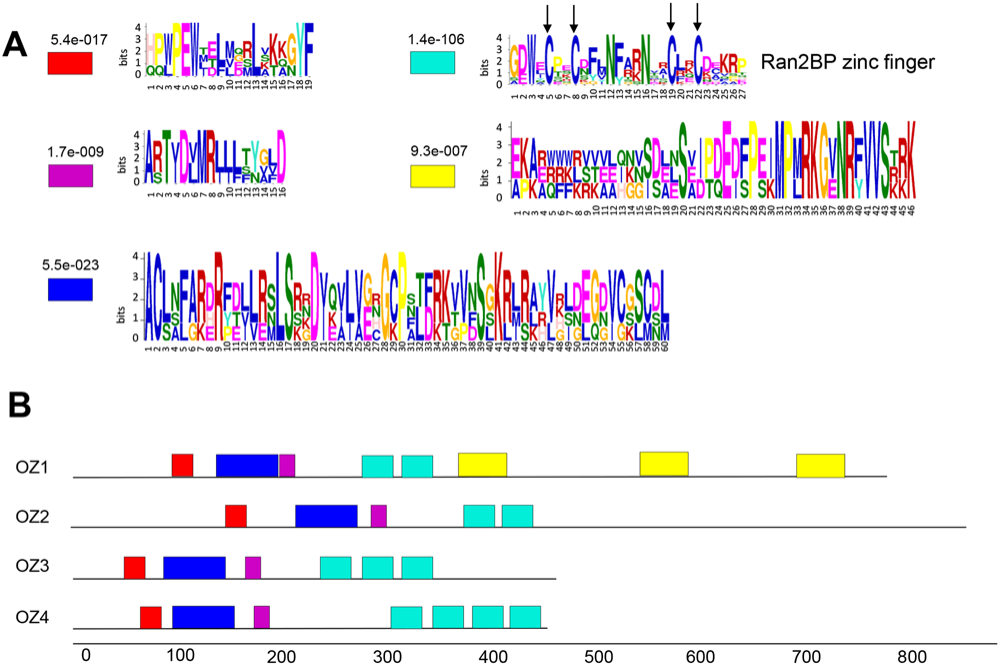
OZ1 and its family members contain multiple domains. (A) motifs detected by MEME prediction for OZ family members. Motif4 is the RanBP2 type Zinc finger domain. Arrows indicate the cysteines characteristic of zinc finger domains. (B) motif locations in the OZ family.

Except for the zinc finger motif, no other annotated domain or motif was found in the OZ1 family. In order to find hidden uncharacterized motifs, motif scanning was performed using MEME against all four members to look for motifs between 15aa to 70aa. Five motifs were returned (Fig. 13). The zinc finger domain has 4 characteristic cysteine residues. As shown in Fig. 13, the zinc finger motif is shared by all four members, but the number of repeats varies. OZ1 and OZ2 (At1g55040) contain two zinc finger motifs, while OZ3 (At1g70650) has three and OZ4 (At1g48570) has four. The regions preceding the zinc finger motifs are relatively highly conserved, briefly spanning 3 distinct domains. The region downstream of the zinc finger domains is quite variable. OZ1 has three repeats of motif 5, which is either missing or poorly conserved in the other members (S2 Fig). Portions of motif 5 were previously identified as three “long repeats” in At5g17790 [28].

**Fig 14:**
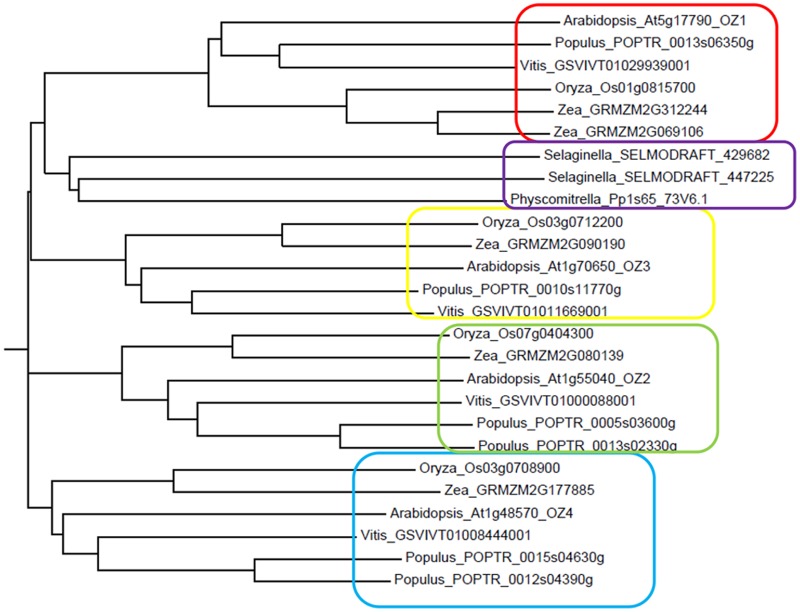
Phylogenetic tree of the OZ family. OZ1 protein sequence was used in a Blast search against protein databases of other plant species. The phylogenetic tree was generated in Clustal Omega (http://www.ebi.ac.uk/Tools/msa/clustalo/) and then visualized by Phylodendron (http://iubio.bio.indiana.edu/treeapp/). Distinct clades were boxed.

We performed homology searches to determine whether orthologs of the four Arabidopsis OZ family members could be detected in other well-characterized plant genomes. We were able to identify putative orthologs for all 4 genes in poplar, grape, rice, and maize. Moss and Selaginella, which exhibit chloroplast RNA editing, also encode OZ-like proteins but with a lower similarity (Fig. 14). We could not detect proteins similar to the OZ family in Chlamydomonas or Volvox, where editing does not occur.

## Discussion

Because a second coding region mutant in *OZ1 w*as not available, we performed transient silencing, transient complementation, and stable complementation to verify the function of OZ1 in editing. Given that VIGS can only knock down gene expression, the editing level of the *OZ1-*dependent sites were reduced but not totally abolished. Introduction of a *35S*::*OZ1* construct into mutant protoplasts or into transgenic plants greatly increased the editing extents of the editing defective sites, demonstrating that the editing defects seen in the *oz1* mutant can be attributed to loss of *OZ1*. The absence of OZ1 results in reduced editing efficiency at most of the affected C targets, but editing of *rpoC1* C488 is increased. Possibly, the loss of *OZ1* results in reduction of sequestration of an editing factor needed for *rpoC1* C488 that is present in limiting amounts when OZ1 is present. If OZ1 is not needed for editing of *rpoC1* C488, its loss could make available more of an unknown editing factor needed for efficient *rpoC1* C488 editing.

Although 14 chloroplast C targets have major loss of editing with nine sites showing no detectable editing by PPE, and editing efficiencies of 16 other editing sites are significantly altered, the *oz1* mutant can survive on sucrose as a yellow seedling and then undergo sufficient chloroplast development to support autotrophic growth. Even in the green, fully photosynthetic leaves, the editing defects are still observed. Most of the sites at which editing is abolished in the *oz1–1* mutant are in non-coding regions or in NADH dehydrogenase genes that are not needed in low light growth chamber conditions. The virescent phenotype could be largely due to the complete loss of editing of *rpoA* C200. Absence of editing at this site also occurs when the PPR editing factor gene *CLB19* is mutated [15], and the phenotype of *oz1–1* is similar to the *clb19* mutant. *rpoA* encodes a subunit of the plastid-encoded RNA polymerase (PEP); loss of editing at this particular site results in defective PEP and yellow seedling phenotype in early developmental stages. However, mutants survive defects in PEP and later partially recover because of the presence of the nuclear-encoded plastid polymerase that can remedy some of the impaired gene expression. The phenotype of *oz1–1* differs from the previously characterized Ds insertion mutant *var3* in At5g17790, which was found to exhibit variegated leaves [28].

**Fig 15:**
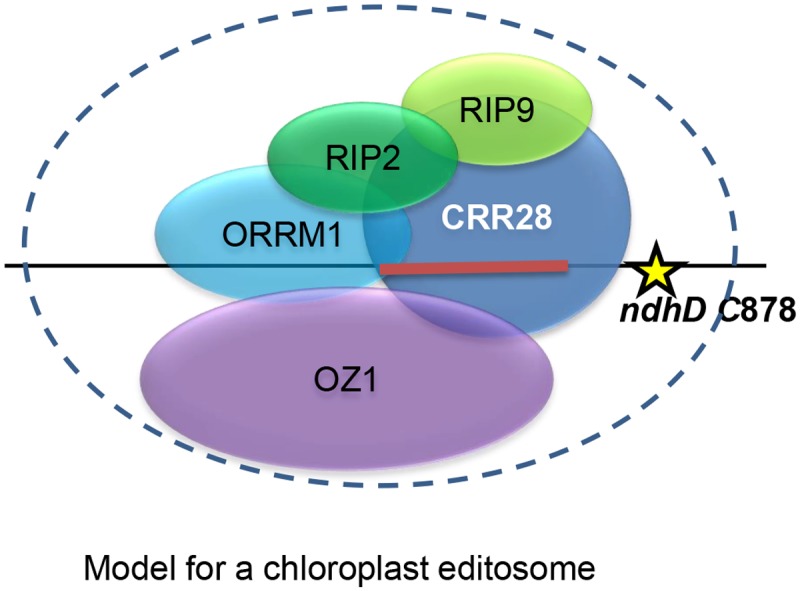
Model of the chloroplast editosome that operates on *ndhD* C878 based on protein-protein interaction data. Data is from yeast two-hybrid assays from this study and prior reports [25,26,31]. The stoichiometry of the components is unknown. The targeted C at position 878 on the *ndhD* transcript is represented by a yellow star, the *cis* element upstream of the target C is indicated by a red bar.

The action of *OZ1* is clearly site-specific, because C targets that reside on the same transcript are differently affected. For example, *ndhD* C878 has a major loss of editing while *ndhD* C383 is barely affected. Which editing sites are affected is likely determined by the editing factors with which OZ1 interacts. C targets that share PPR recognition factors are similarly affected in the *oz1* mutant. In Y2H assays, OZ1 binds to CRR28 and OTP82, PPR proteins that are required for editing of sites that also require OZ1. Furthermore, OZ1 interacts with ORRM1, and all 14 severely affected chloroplast sites are also affected in the *orrm1* mutant. OZ1 interacts with RIP1, though the interaction is not as strong as that with ORRM1. We did not observe direct interaction of OZ1 with RIP2 or RIP9. However, ORRM1 can bind to RIP1 and RIP2. Previously we reported interaction between the RIP-RIP domain of ORRM1 and CRR28 and OTP82 [25]. Another group determined that RIP2 and RIP9 interact with CRR28 [31]. RIP2 and RIP9 have been reported to interact with PPO1, protoporphyrinogen IX oxidase 1, which is required for efficient editing of a number of chloroplast sites [31]. However, PPO1 does not interact with CRR28 or other PPR editing factors, and it is presently unknown whether PPO1 also interacts with either ORRM1 or OZ1 [31]. All of the interaction data, taken together, is consistent with the presence of multi-component editing complexes that contain ORRM1, OZ1, and at least one RIP protein and a PPR protein, at unknown stoichiometry. An example of the model for the editosome acting upon *ndhD* C878, drawn according to the yeast two-hybrid data, is shown in Fig. 15. Some complexes are also likely to contain PPO1, but we cannot place this protein into our diagram until its interaction with ORRM1 and OZ1 is investigated in the future.

Three proteins that share high similarity with OZ1 are also predicted to be targeted to organelles according to Target P (S2 Table). OZ1 contains three long repeats at its C terminus; these domains of unknown function are less conserved in other family members. The only well-documented and most significant domain found in this family is the Ran binding protein 2 type zinc finger motif, which is a conserved 30-amino-acid consensus (X_2_GDWICX_2_CX_3_NFARRX_2_CXRCX_2_-PRPEX_2_; pFAM00641) characterized in RAN binding protein 2 (RanBP2) and other nucleoporins. OZ1 contains two tandem RanBP2 zinc-finger domains while other members have various number of this domain. It is highly possible that other members of the OZ family have similar role in chloroplast or mitochondrial RNA editing. Such redundancy could explain the residual editing for some sites in the *oz1* mutant Chloroplast RNA editing *in vitro* has been shown to be Zn^2+^ dependent [32]. Zinc binding is characteristic of cytidine deaminases, and the DYW domain, which contains cytidine deaminase motifs and is present on a subset of PPR protein editing factors, has been shown to bind zinc ions [16,23]. The requirement for zinc in plant organelle RNA editing has been thought to be due to the need for cytidine deaminase activity. The discovery of OZ1 implicates the OZ family as another possible source of the zinc requirement for editing to occur. Further experiments will be needed to determine whether the zinc fingers present in the OZ family actually bind zinc and whether they are important in RNA and/or protein binding in the RNA editosome.

## Material and Methods

### Mutant lines and phenotyping

The T-DNA insertional *A*. *thaliana* Columbia ecotype mutant SAIL_358_H03 in the *OZ1* gene was obtained from the Arabidopsis Biological Resource Center (https://abrc.osu.edu/). After 3 days of stratification, the seeds were placed onto petri dishes containing Murashige-Skoog medium in a 25^o^ room with 14 hour day length. Mutant plants and the wild-type siblings were then transferred into soil after 7 weeks on tissue culture medium. Leaves from 4 week- old and 8 week- old plants were collected for further analysis.

### Plasmid constructs and oligonucleotides

The ORRM1 coding sequence was cloned using primer pair ORRM1_1F and ORRM1_R_WO from previous constructs. The sequence was first cloned into PCR8/GW/TOPO (Life Technologies, Carlsbad, CA), and then shuttled into a modified PBI121 vector with a 3XFLAG-strepII C-terminal tag. Alternatively, coding sequence of the ORRM1 mature form (without the predicted 54aa transit peptide) was fused to a N terminal 3XFLAG-strepII tag sequence and an artificial transit peptide sequence from RecA in an overlapping PCR using primer pairs RecA_F, RecA_R, 3FS_F, 3FS_R, ORRM1_163F and ORRM1_R. This chimeric gene was cloned into PCR8 vector first and then the PBI121 vector using LR ClonaseII (Life Technologies, Carlsbad, CA). The C-terminal tag on the vector was eliminated by the endogenous stop codon of ORRM1 in the sequence. A *RecA-RRM* construct was described previously. The OZ1 coding sequence was cloned using primer pair OZ1_F and OZ1_R from *A*. *thaliana* cDNA. The PCR product was first ligated to PCR8/GW/TOPO and then transferred into the destination vectors pSAT4a and pAUL13 [33] to create a transient expression construct and a stable complementation construct, respectively.

All oligonucleotides used in this study are shown in S3 Table.

### Protoplast complementation

Protoplasts from *orrm1* or *oz1* mutants were prepared following the protocol from Jen Sheen’s lab [27]. Light green leaves from the *oz1* mutant were used for protoplast preparation. 10 μg of plasmid DNA was used to transfect 2x10^4^ cells. The transfected protoplasts were incubated in the dark at room temperature for 3 days (*orrm1* complementation) or 1 day (*oz1* complementation) before harvest.

### Virus-induced gene silencing

An *OZ1* gene-specific region was selected from the CATMA database [34] and amplified using primer pair OZ1_VIGS_F and OZ1_VIGS_R. The fragment was first integrated into PCR8/GW/TOPO and then into the silencing vector PTRV2/GW/GFP by an LR reaction. Agrobacteria harboring the silencing construct were used to infiltrate 2 week-old Arabidopsis seedlings that expressed GFP driven by 35S promoter as previously described [7]. 5 weeks after infiltration, silencing efficiency was monitored by the expression of the co-silenced GFP in each individual. Silenced plants, which exhibited a dark red color from stem to leaf under UV light, were collected for further analysis.

### Generation of transgenic plants

The 3XFLAG-strepII-ORRM1ΔCTP and OZ1 constructs in PBI121 and PAUL13, respectively, were used to transform the Agrobacterium GV3101 strain. A standard root transformation protocol was followed to transform mutant *orrm1* roots. The roots were first induced on Callus Inducing Medium (CIM) for 2 days and then infected with Agrobacteria in liquid media [35]. Roots were incubated on CIM for another 2 days until they were overgrown by Agrobacteria and then bacteria were removed by several washing steps with liquid CIM containing timentin and carbenicillin. Roots were then cut into 0.5mm pieces and put onto Shoot Inducing Medium (SIM) containing 100mg/L Basta for selection. After the shoots grew out, they were removed from the calli and transferred onto a Root Inducing Medium (RIM) [35]. Fully grown transgenic plants with healthy roots were then transferred into soil. Because the root transformation was not successful with the *oz1* mutant, a standard floral dip protocol was used to transform a heterozygous plant.

### Co-immunoprecipitation

10 g of leaves of each line were ground in liquid nitrogen into fine powder. Total leaf protein was extracted using grinding buffer (150mM NaCl, 50mM Tris-HCl pH7.4, 1mM EDTA, 0.2%NP-40 and 1x cocktail protease inhibitor (Sigma-Aldrich, St. Louis, MO). The extract was cleared by 13,000 rpm centrifugation, 0.45 μm filtration and then 30 minutes incubation with unconjugated agarose beads (Vector Laboratories, Burlingame, CA) to minimize non-specific binding. 200 μl anti-FLAG agarose resins (Sigma-Aldrich, St. Louis, MO) were first blocked with 4%BSA before 2 hours incubation with around 30 μg pre-cleared protein extract. A washing step was done using washing buffer (150mM NaCl, 50mM Tris-HCl pH7.4, 1mM EDTA, 0.2% NP-40). The IP was eluted with elution buffer (2M MgCl_2_, 50mM Tris-HCl ph8.0, 150mM NaCl, 0.5%CHAPS). The final sample was prepared using SDS-PAGE sample prep kit (Thermo Scientific, Waltham, MA).

### Immunoblotting, silver staining and mass spectrometry

1% of the IP samples were loaded onto an Any kD MINI-PROTEAN TGX precast gel (Bio-Rad, Hercules, CA) followed by standard procedures of immunoblotting. α-FLAG-M2-HRP (Sigma-Aldrich, St. Louis, MO) was used to detect FLAG-tagged proteins. 10% of the IP samples were separated on a 10% polyacrylamide gel before being subjected to silver staining compatible to mass spectrometry. Co-purifying proteins with FLAG-tagged ORMM1 were identified by tandem mass spectrometry using a nanoLC-LTQ-Orbitrap instrument, followed by database searching with MASCOT against TAIR10 [36].

### RNA editing extent measurement

DNA contaminants were removed from RNA samples by TURBO DNase (Life Technologies, Carlsbad, CA). The cDNA was reverse transcribed from the RNAs using the pooled reverse primers as previously described [7]. PCR products harboring the editing sites were either bulk Sanger-sequenced or subjected to PPE assay [29,37].

### Yeast two-hybrid assay

Mature OZ1 coding sequence (without the N-terminal predicted 33 aa transit peptide) was amplified using primer pair OZ1_100F and OZ1_R from cDNA and cloned into PCR8/GW/TOPO (Life Technologies, Carlsbad, CA). Mature RIP2 and RIP9 coding sequences were amplified using primer pairs RIP2_133F and RIP2_R, RIP9_175F and RIP9_R from *A*. *thaliana* cDNA, respectively. PCR products were first cloned into PCR8/GW/TOPO and then pGADT7GW and pGBKT7GW destination vectors through homologous recombination by LR clonaseII (Life Technologies, Carlsbad, CA). RIP1, RARE1, CRR28, OTP82, ORRM1 constructs produced for Y2H assays were previously described [7]. Empty vectors were used as negative controls. Two mating types of the PJ69–4 yeast strain, a and α, were used. Single transformants were obtained by transformation while double transformants were produced through mating. Yeast harboring testing pairs were grown in leucine and tryptophan deficient media overnight before they were diluted with water to OD_600_ 0.5, 0.05, or 0.005. 10 μl of each dilution was spotted onto leucine-, tryptophan-, histidine-, adenine-deficient media plates. Growth results were collected after 3 days incubation at 30°C.

### Subcellular localization in protoplasts

The first 100 codons of OZ1 was amplified from cDNA with primers listed in S3 Table, and cloned into PCR8/GW/TOPO (Life Technologies, Carlsbad, CA). The fragment was subsequently transferred to the pEXSG-YFP Gateway destination vector [38] by recombination using LR Clonase II (Life Technologies, Carlsbad, CA), creating a gene encoding a YFP fusion protein driven by a tandem CaMV 35S promoter. Protoplasts were prepared from leaves of 3-week old Arabidopsis accession Ler and transfected as described above. Images were acquired 3 days after transfection using a Zeiss 710 confocal microscope at the Cornell Biotechnology Resource Center (BRC).

## Supporting Information

S1 Fig Epitope-tagged ORRM1 is functional in a transient complementation assay. **(A)** Schematic diagram of the C- terminal and N-terminal epitope-tagged ORRM1 constructs. (B) C-terminal epitope-tagged ORRM1 fails to complement editing defect in a transient complementation assay. Editing extent is examined by PPE. (C) N-terminal tagged ORRM1 can enhance editing of matK C640 in *orrm1* protoplasts. Star indicates significant difference (P<0.01) with the non-transfected control. Not transfected: untreated *orrm1* protoplasts; ORRM1–3FS: *orrm1* protoplasts transfected with a construct expressing C-terminal epitope-tagged ORRM1; RecA-RRM: *orrm1* protoplasts transfected with a construct expressing RecA transit peptide fused with the RRM motif of ORRM1; RecA-3FS-mORRM1: *orrm1* protoplasts transfected with this construct. E, edited product; U, unedited product; O, oligonucleotide.
(TIF)

S2 Fig OZ1 (At5g17790) protein sequence and predicted motifs. (TIF)

S1 Table Summary of the MS/MS based identification of AT5G17790.1 in co-immunoprecipitates with ORRM1. (XLSX)

S2 Table Localization prediction for OZ family members by TargetP. (XLSX)

S3 Table Oligonucleotides used in this study. (XLSX)
