# Supplementary figures and images for: A Zinc Finger Motif-Containing Protein Is Essential for Chloroplast RNA Editing
Source: PLoS Genet. 2015 Mar 13;11(3):e1005028. doi: 10.1371/journal.pgen.1005028 (PMC4359148; doi:10.1371/journal.pgen.1005028)

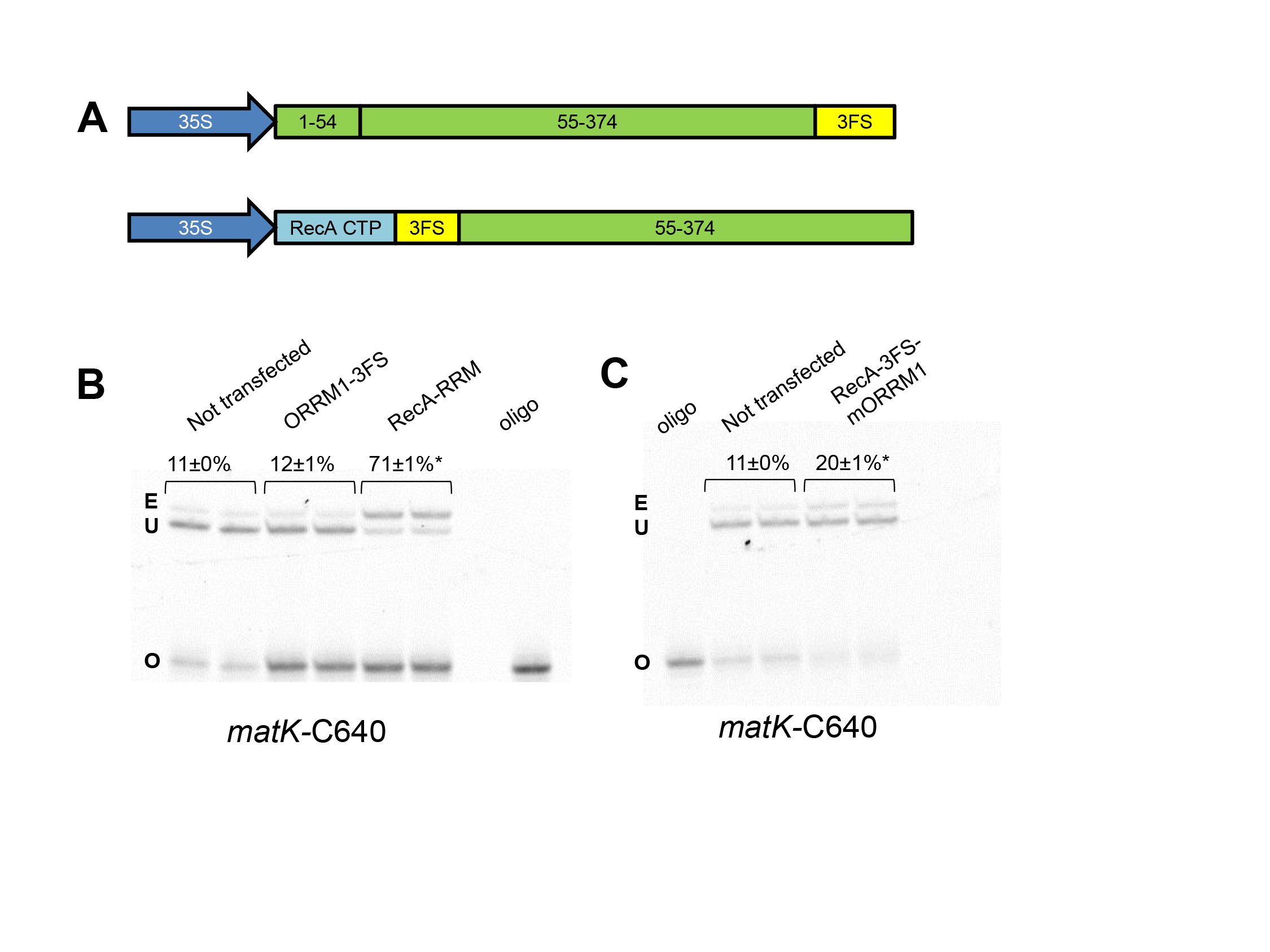

Supplement: S1 Fig — (A) Schematic diagram of the C- terminal and N-terminal epitope-tagged ORRM1 constructs. (B) C-terminal epitope-tagged ORRM1 fails to complement editing defect in a transient complementation assay. Editing extent is examined by PPE. (C) N-terminal tagged ORRM1 can enhance editing of matK C640 in orrm1 protoplasts. Star indicates significant difference (P<0.01) with the non-transfected control. Not transfected: untreated orrm1 protoplasts; ORRM1–3FS: orrm1 protoplasts transfected with a construct expressing C-terminal epitope-tagged ORRM1; RecA-RRM: orrm1 protoplasts transfected with a construct expressing RecA transit peptide fused with the RRM motif of ORRM1; RecA-3FS-mORRM1: orrm1 protoplasts transfected with this construct. E, edited product; U, unedited product; O, oligonucleotide. (TIF) [file pgen.1005028.s001.tif]

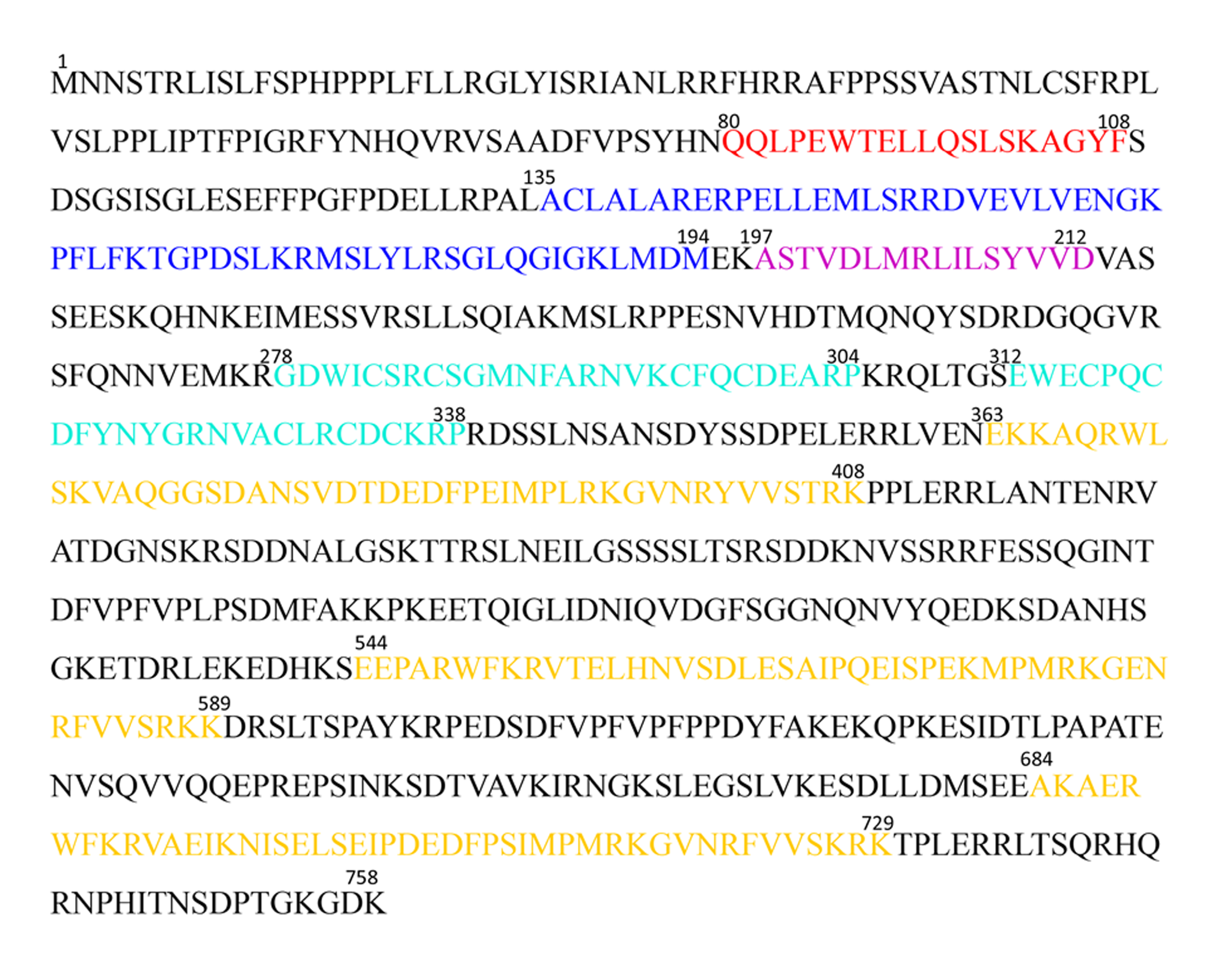

Supplement: S2 Fig — (TIF) [file pgen.1005028.s002.tif]
